# Decision-Making for the Autonomous Navigation of Maritime Autonomous Surface Ships Based on Scene Division and Deep Reinforcement Learning

**DOI:** 10.3390/s19184055

**Published:** 2019-09-19

**Authors:** Xinyu Zhang, Chengbo Wang, Yuanchang Liu, Xiang Chen

**Affiliations:** 1 Key Laboratory of Maritime Dynamic Simulation and Control of Ministry of Transportation, Dalian Maritime University, Dalian 116026, China; 2 Marine Engineering College, Dalian Maritime University, Dalian 116026, China; 3 Department of Mechanical Engineering, University College London, Torrington Place, London WC1E 7JE, UK; yuanchang.liu@ucl.ac.uk; 4 Department of Civil Environmental and Geomatic Engineering, London WC1E 6BT, UK; xiang.chen.17@ucl.ac.uk

**Keywords:** decision-making, autonomous navigation, collision avoidance, scene division, deep reinforcement learning, maritime autonomous surface ships

## Abstract

This research focuses on the adaptive navigation of maritime autonomous surface ships (MASSs) in an uncertain environment. To achieve intelligent obstacle avoidance of MASSs in a port, an autonomous navigation decision-making model based on hierarchical deep reinforcement learning is proposed. The model is mainly composed of two layers: the scene division layer and an autonomous navigation decision-making layer. The scene division layer mainly quantifies the sub-scenarios according to the International Regulations for Preventing Collisions at Sea (COLREG). This research divides the navigational situation of a ship into entities and attributes based on the ontology model and Protégé language. In the decision-making layer, we designed a deep Q-learning algorithm utilizing the environmental model, ship motion space, reward function, and search strategy to learn the environmental state in a quantized sub-scenario to train the navigation strategy. Finally, two sets of verification experiments of the deep reinforcement learning (DRL) and improved DRL algorithms were designed with Rizhao port as a study case. Moreover, the experimental data were analyzed in terms of the convergence trend, iterative path, and collision avoidance effect. The results indicate that the improved DRL algorithm could effectively improve the navigation safety and collision avoidance.

## 1. Introduction

Recently, marine accidents have been frequently caused by human factors. Based on the statistics from the European Maritime Safety Agency (EMSA), in 2017, there were 3301 casualties and accidents at sea, with 61 deaths, 1018 injuries, and 122 investigations initiated. In these cases, human error behavior represented 58% of the accidents and 70% of the accidents were related to shipboard operations. In addition, the combination of collision (23.2%), contact (16.3%), and grounding/stranding (16.6%) shows that navigational casualties represent 56.1% of all casualties with ships [1]. The important purpose of maritime autonomous surface ships (MASSs) research is to reduce the incidence of marine traffic accidents and ensure safe navigation. Therefore, safe driving and safe automatic navigation have become urgent problems in the navigation field. Future shipping systems will rely less and less on people, and the efficiency of ship traffic management is getting higher and higher. It further highlights the shipping industry’s need for MASSs and their technology.

At present, many foreign enterprises and institutions have completed the concept design of MASS and the port-to-port autonomous navigation test [2,3,4]. However, for China, from 2009 to 2017, domestic organizations, such as the First Institute of Oceanography of the State Oceanic Administration, Yun Zhou Intelligent Technology Co., Ltd. Zhuhai, China, Ling Whale Technology, Harbin Engineering University, Wuhan University of Technology, and Huazhong University of Science and Technology, have conducted research on unmanned surface vessels (USVs). There are some differences in autonomous navigation technology of MASSs compared to USVs.

1. First, the molded dimension of a MASS is larger. Research on the key technologies of USVs pays more attention to motion control. However, for MASSs, navigation brains that can make autonomous navigation decisions are needed more.
2. Second, the navigation situation of a MASS is complex and changeable, and its maneuverability is slow to respond. Therefore, it is necessary to combine scene division with adaptive autonomous navigation decision-making in order to achieve safe decision-making for local autonomous navigation.

Owing to these differences, the autonomous navigation decision-making system is the core of a MASS, and its effectiveness directly determines the safety and reliability of navigation, playing a role similar to the human “brain.” During the voyage, the thinking and decision-making process is very complex. After clarifying the destinations that need to be reached and obtaining the global driving route, it is necessary to generate a reasonable, safe, and efficient abstract navigation action (such as acceleration, deceleration, and steering) based on the dynamic environmental situation around the ship. The “brain” needs to rapidly and accurately reason based on multi-source heterogeneous information such as the traffic rule knowledge, driving experience knowledge, and chart information stored in its memory unit. In this paper, we only train navigation strategies by learning relative distance and relative position data. We assumed the following perception principles.

**Figure 1:**
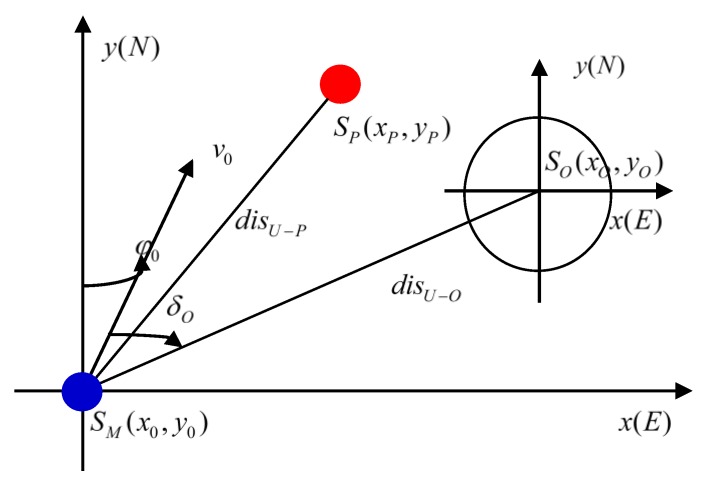
Schematic diagram of perception.

The input information of the autonomous navigation decision-making system is multi-source heterogeneous, including real-time sensing information from multiple sensors and various a priori pieces of information. In the environmental model, the MASS sensor detects the distance and relative azimuth between the MASS and the obstacle. Figure 1 illustrates the MASS perception. In the figure, the geographical coordinate of MASS is $S_{M}(x_{0},y_{0})$; speed is $v_{0}$; ship course is $\varphi_{0}$; geographical coordinate of the static obstacle is $S_{O}(x_{o},y_{o})$; relative bearing of the MASS and obstacles is $\delta_{0}$; $S_{P}(x_{p},y_{p})$ is the position of the target point; $dis_{M-P}$ is the distance between MASS and target point; and $dis_{M-O}$ is the distance between MASS and obstacle. Among these symbols, the subscripts in the symbols are as follows: “M” is for the MASS, “P” is for target point, and “O” is for obstacle.

The current environmental status information can be expressed as $obs_{t}={\left[v_{0},\varphi_{0},\delta_{0},dis_{M-P},dis_{M-O}\right]}^{T}$. The algorithm not only acquires the current state $obs_{t}$ of the obstacle, but also obtains the historical observation state ($obs_{t-i},i\in 1,\cdots ,T_{P}$), where $T_{P}$ is the total length of the observation memory. The data input for the final training is $X_{Perception}(t)={\left[obs_{t}\;obs_{t-1}\;\cdots \;obs_{t-T_{P}}\right]}^{T}$. Therefore, the input of the high-level driving decision-making system at time *t* can be expressed as follows:

(1) $$\begin{array}{c} X_{Perception}(t)={\left[obs_{t}\;obs_{t-1}\;\cdots \;obs_{t-T_{P}}\right]}^{T}\\ =\left[\begin{array}{cccc} v_{t} & \varphi_{t} & \delta_{t} & \begin{array}{cc} dis_{t^{M-P}} & dis_{t^{M-O}} \end{array}\\ v_{t-1} & \varphi_{t-1} & \delta_{t-1} & \begin{array}{cc} dis_{t-1^{M-P}} & dis_{t-1^{M-O}} \end{array}\\ \vdots & \vdots & \vdots & \begin{array}{cc} \vdots & \vdots \end{array}\\ v_{t-T_{P}} & \varphi_{t-T_{P}} & \delta_{t-T_{P}} & \begin{array}{cc} dis_{t-T_{P}{}^{M-P}} & dis_{t-T_{P}{}^{M-O}} \end{array} \end{array}\right] \end{array}$$

Learning from the decision-making of the officer on the voyage, this research proposes a hierarchical progressive navigation decision-making system, which mainly includes two sub-modules: a scene division module and a navigation action generation module. The main contributions of this paper are as follows:

1. We exploit ontology and the principle of divide and conquer to construct the navigation situation understanding model of a MASS, and divide the situation of MASS navigation into scenes based on the International Regulations for Preventing Collisions at Sea (COLREGS).
2. Aiming at the problem of local path planning and collision avoidance decision-making, a method of autonomous navigation decision-making for MASSs based on deep reinforcement learning is proposed, in which the reward function of multi-objective optimization is designed, which consists of safety and approaching target points.
3. An artificial potential field is added to alleviate the problem of easy-to-fall-into local iterations and slow iterations of autonomous navigation decision-making algorithms based on deep reinforcement learning.
4. Simulation results based on Python and Pygame show that the Artificial Potential Field-Deep Reinforcement Learning (APF-DRL) method has better performances than the DRL method in both autonomous navigation decision-making and algorithm iteration efficiency.

The remaining sections of the paper are organized as follows. Related works are presented in Section 2. The scene division module is presented in Section 3. The autonomous navigation decision-making module is presented in Section 4. The simulation results and algorithm improvement are presented in Section 5. The paper is concluded in Section 6. 

## 2. Related Work

The autonomous navigation decision-making system of a MASS plays the role of the “navigation brain.” The problem to be solved is to determine the best navigation strategy based on environmental information. At present, related works mainly focus on the ship’s intelligent collision avoidance algorithms in specific environments.

For the study on intelligent collision avoidance and path planning of ships, the existing models mainly contain knowledge-based expert systems, fuzzy logic, artificial neural networks, intelligent algorithms (genetic algorithms, ant colony algorithms, etc.). In addition, a ship collision avoidance system based on the general structural model of the expert system has been established [5]. Moreover, a comprehensive and systematic study has been performed for the whole process of ship collision avoidance, and a mathematical model for the safe passing distance, pressing situation, and ship collision risk has been established. Fan et al. [6] combined the dynamic collision avoidance algorithm and tracking control, and as such, a dynamic collision avoidance control method in the unknown ocean environment is presented. A novel dynamic programming (DP) method was proposed to generate the optimal multiple interval motion plan for a MASS by Geng et al. [7]. The method provided the lowest collision rate overall and better sailing efficiency than the greedy approaches. Ahn et al. [8] combined fuzzy inference systems with expert systems for collision avoidance systems. They proposed a method for calculating the collision risk using a neural network. Based on the distance to closest point of approach (DCPA) and the time to closest point of approach (TCPA), the multi-layer perceptron (MLP) neural network was applied to the collision avoidance system to compensate for the fuzzy logic. Hua [9] optimized the shortest path and minimum heading of the local path and designed the surface planning of the surface unmanned submarine under the constraints of the close distance meeting model of the ship and 1972 International Collision Avoidance Rules. The target genetic algorithm realized the intelligent collision avoidance of unmanned boats through simulation. Ramos et al. [10] presented a task analysis for collision avoidance through hierarchical task analysis and used a cognitive model for categorizing the tasks, which explored how humans can be a key factor for successful collision avoidance in future MASS operations. The results provided valuable information for the design stage of a MASS. For the study on path-following and control of autonomous ships, a novel translation–rotation cascade control scheme was developed for path-following of an autonomous underactuated ship by Wang et al., and in the case of disturbance, the autonomous underactuated ship was controlled, and the trajectory point guidance was used for precise tracking and autonomous navigation [11,12,13].

The abovementioned models usually assume complete environmental information. However, in an unknown environment, prior knowledge of the environment is difficult to acquire. It is difficult to form a complete and accurate knowledge base, and the rule-based algorithm makes it difficult to cope with various situations. Therefore, in many practices, the system needs to have a strong adaptive ability to adjust to the uncertain environment. Recently, deep reinforcement learning combined with deep neural network models and reinforcement learning have made significant progress in the field of autonomous driving, such as unmanned surface vehicles (USV), unmanned aerial vehicles (UAV), and unmanned ground vehicles (UGV). Tai et al. [14] combined deep learning and decision-making processes into a highly compact, fully connected network with raw depth images as the input and the generated control commands as the outputs to achieve model-free obstacle avoidance behavior. Long et al. [15] proposed a novel end-to-end framework for generating effective reactive collision avoidance strategies for distributed multi-agent navigation based on deep learning. Panov et al. [16] proposed an approach for using a neural network to perform the path planning on the grid and initially realize it based on deep reinforcement learning. Bojarski et al. [17] used convolutional neural networks for end-to-end training driving behavioral data, mapping the raw pixels from a single-front camera directly to the steering commands for unmanned vehicle adaptation path planning. The performance of the model and results of learning were better than the traditional model, but the only improvement was that the model was less interpretable. Cheng et al. [18] proposed a simple deep reinforcement learning obstacle avoidance algorithm based on the deep Q learning network using a convolutional neural network to train the ship sensor image information. The interaction with the environment was included by designing the incentive function in reinforcement learning. The maximum expected value of the cumulative return was obtained, and the optimal driving strategy of the underactuated USV was derived. However, improvement in this area is needed to increase the complexity of the verification environment and dynamic obstacle environment. Compared with Cheng et al. [18], the different and better aspects of our paper are: First, we used long short-term memory (LSTM) to store historical decisions for autonomous navigation by improving the iterative effectiveness. Second, the method used in this paper learns the ship navigation state data, including relative azimuth and relative distance, to improve the accuracy and effectiveness of autonomous navigation of a MASS. Third, in order to solve the problem of easy-to-fall-into local iteration and slow iteration speed, we added a gravitational field to improve deep reinforcement learning with the target point as the potential field center. In summary, the deep reinforcement learning achieved self-adaptation to an unknown environment by self-training various experiences and using high-dimensional inputs such as raw images or environmental states.

However, few experts currently apply deep reinforcement learning to MASS intelligent navigation. Taking advantage of these, this paper uses deep reinforcement learning to solve the problem of autonomous navigation decision-making for a MASS. Learning from the decision-making of the officer on the voyage, this research proposes a hierarchical progressive navigation decision-making system, which mainly includes two sub-modules: a scene division module and an autonomous navigation action generation module.

## 3. Scene Division Module for a MASS

The scene division mainly organizes and describes the multi-source heterogeneous information in the driving scene with the Prolog language [19]. This research uses the ontology theory and the principle of divide and conquer to divide navigation environment into entities and attributes. Entity classes are used to describe the objects of different attributes, including chart entity, obstacle entity, ego ship, and environment. Attribute classes are used to describe the semantic properties of an object, including position attributes and orientation attributes.

Ontology is a philosophy concept, which studies the nature of existence. Ontology can be classified into four types: domain, general, application, and representation [20]. A complete marine transportation system is a closed-loop feedback system consisting of “human–ship–sea–environment.”

The entity class is categorized into four sub-entity classes: chart entity, obstacle entity, MASS entity (egoship), and environmental information. Chart entity includes point entity, line entity, and area entity, where point entity refers to navigational aids and line entity refer to reporting lines. The area entity includes seapart, channel, boundary, narrow channel, divider, anchorage, junction, and segment. Obstacle entities include static obstacle and dynamic obstacle, where static obstacle entities are divided into rocks, wreck, and boundary; and dynamic obstacle entities include ships (vessel), floating ice, and marine animal. A MASS entity (egoship) is used to describe its own state information. Environmental information includes height, sounding, nature of the seabed, visibility, and disturbance.

The relationship between the MASS and the obstacles can be divided into binary relationships: MASS and static obstacles (abbreviated as ES), and MASS and dynamic obstacles (abbreviated as ED). In the azimuthal relationship, the abbreviations are as follows: HO is the head-on encounter, OT is the overtaking encounter, and CR is the crossing encounter. The MASS ontology model relationship attribute is presented in Table 1.

**Figure 2:**
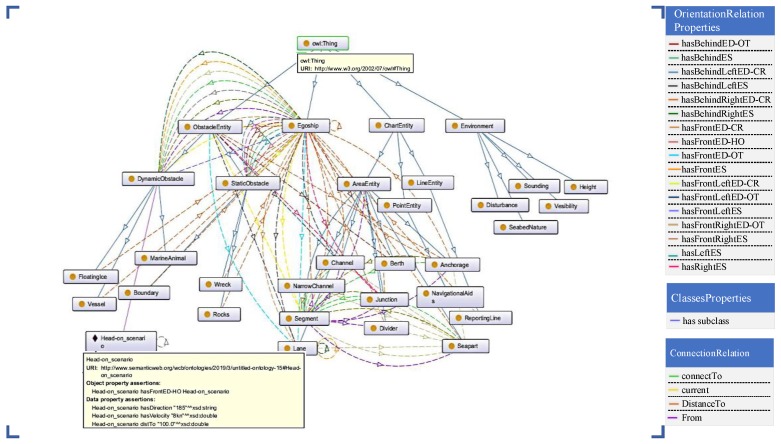
Ontology conceptual model diagram of the navigation scene.

The scene ontology model corresponding to the relationship property of the ontology model of MASS in Table 1 is established. Figure 2 shows the ontology conceptual model of the MASS navigation scene.

Combining the COLREGS, the navigation scenes of Egoship−StaticObstacle and Egoship−DynamicObstacle are divided into six scenes: hasFront, hasFrontLeft, hasFrontRight, hasBehind, hasBehindLeft, hasBehindRight. Then, the scenes corresponding to Egoship−DynamicObstacle are divided into the HO sub-scenario, the CR sub-scenario, the OT sub-scenario, and the mixed sub-scenarios. However, this paper mainly analyses the scene division between MASS and dynamic obstacle. So six scenes is become hasFrontED, hasFrontLeftED, hasFrontRightED, hasBehindED, hasBehindLeftED, hasBehindRightED.

hasFrontED: 15π8∼π8, including HO, OT, and CR.
hasBehindED: 5π8∼11π8, including OT.
hasFronntLeftED: 3π2∼15π8, including CR and OT.
hasFrontRightED: π8∼π2, including CR and OT.
hasBehindLeeftED: 11π8∼3π2, only including CR.
hasBehindRightED: π2∼5π8, only including CR.

**Figure 3:**
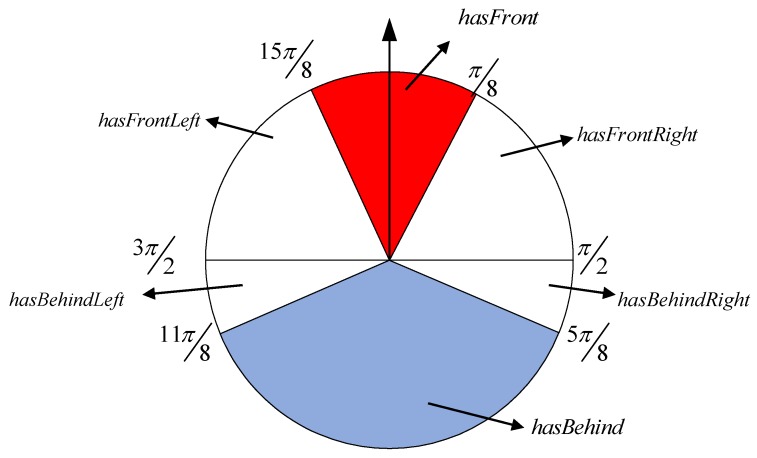
A quantitative map of the scene division based on the International Regulations for Preventing Collisions at Sea (COLREGS).

In summary, the visual display of the six scenes is shown in Figure 3.

## 4. Autonomous Navigation Decision-Making Module for a MASS

Deep reinforcement learning is a combination of deep learning and reinforcement learning. In this paper, Q-learning was combined with a neural network to establish the autonomous navigation decision-making model based on deep Q-learning. The deep Q-learning algorithm uses an empirical playback algorithm whose basic core idea is to remember the historical information that the algorithm performs in this environment. In practice, the number of environmental states and behavioral states is extremely large and it is necessary to adopt a neural network for generalization. Therefore, LSTM was selected as the Q network. The core concepts of LSTM are cell state and “gate” structure. Cell state is equivalent to the path of information transmission such that information can be transmitted in sequence. This can be thought of as the “memory” of the network. LSTM network has three control gates: forget gate, input gate, and output gate. The forget gate determines which environmental states and behavioral states from the last cell state to continue to pass through the current cell. The input gate controls whether a new datum could flow into the memory and updates the cell state. The output gate decides which part of the Q value to be exported as output [21]. The three control gates weaken the short-term memory effect and regulate the predicted Q value corresponding to multiple autonomous navigation actions in the current state.

**Figure 4:**
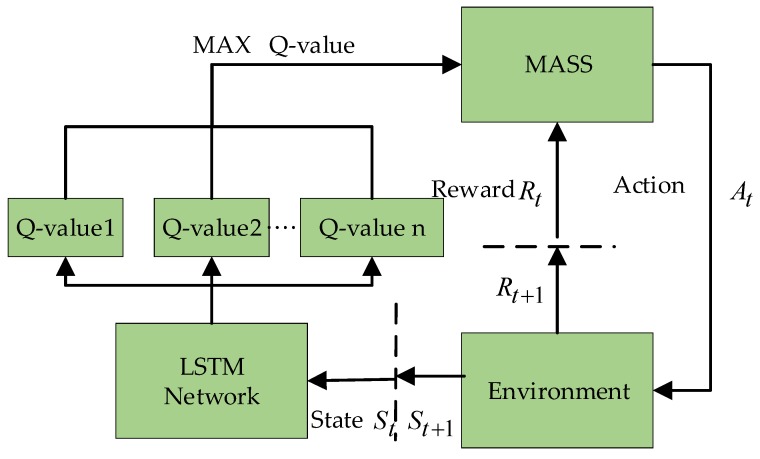
Schematic of the autonomous navigation decision-making of a maritime autonomous surface ship (MASS) based on deep reinforcement learning (DRL).

The mathematical nature of reinforcement learning can be regarded as a Markov decision process (MDP) in discrete time. A Markov decision process is defined using the following five-tuple, (S,A,$P_{a}$,$R_{a}$,γ). S represents the finite state space in which the MASS is located. A represents the behavioral decision space of MASS, i.e., a collection of all the behavioral spaces of the MASS in any state, such as left rudder, right rudder, acceleration, and deceleration. $P_{a}(s,s^{\prime })=P(s^{\prime }|s,a)$ is the conditional probability that represents the probability that the MASS will reach next state $s^{\prime }$ under state s and action a. $R_{a}(s,s^{\prime })$ is a reward function representing the stimulus that the MASS takes from state s to state $s^{\prime }$ under action a. γ∈(0,1) is the discount factor of the stimulus, and the discounting at the next moment is determined according to a factor [22,23]. Figure 4 displays the schematic of the autonomous navigation decision-making of the MASS based on deep reinforcement learning. In the memory pool, the current state of the observed MASS is taken as the input of the neural network. The Q value table of the action that can be performed in the current state is the output, and the behavioral strategy corresponding to the maximum Q value is learned through training.

### 4.1. Representation of Behavioral Space

After setting the initial and target points, the MASS is considered as a particle in the simulation. In a real navigation process, the autonomous navigation of the MASS is a continuous state, following which, observation behavior O needs to be generalized into discrete action $\hat{A}=Generalization(A^{\prime },O)$. Generally, the search action of a MASS includes the four discrete actions of up, down, left, and right. When the environment has a corner, the search behavior in the diagonal direction is increased. Centering on the mass of the MASS, the actual motion space model A is defined as eight discrete actions, $up,\;down,\;left,\;right,\;up_{-45^{\circ }},\;up_{+45^{\circ }},\;down_{-45^{\circ }},\;down_{+45^{\circ }}$, namely, the matrix of Equation (2).

(2) $$A=\left[\begin{array}{cccccccc} -1,1 & 0,1 & 1,1 & -1,0 & 1,0 & -1,-1 & 0,-1 & 1,-1 \end{array}\right]$$

### 4.2. Design of the Reward Function

In the reinforcement learning system of a MASS, the activation function plays an important role in evaluating the effectiveness of the behavioral decision-making and safety of obstacle avoidance. It has a search-oriented role. The goal of reinforcement learning is to obtain the search strategy that gives the highest return value in the driverless process. The reward function consists of safety, comfort, and arrival target points. When designing the reward function, the following elements should be maximally considered [18].

(1) Approach to the target point: The search behavior of the autonomous navigation decision-making made in an uncertain environment should bring the MASS closer to the target point. A value close to the incentive function will choose the reward; otherwise it will be punished:
  (3) $$R_{distance}=-\lambda_{distance}\sqrt{{\left(x-x_{goal}\right)}^{2}+{\left(y-y_{goal}\right)}^{2}}$$
(2) Safety: In the deep Q-learning algorithm model, the unknown environment is divided into a state space, which is divided into a safe state area and an obstacle area. The system should be selected in the local area without the obstacle to sailing. An action search strategy and “early, clear, big-amplitude” is used to avoid obstacles. Thus, in the reward function, the penalty value is added to the behavior close to the obstacle, and the reward value is increased:
  (4) $$R_{collisions}=-\lambda_{collisions}\begin{array}{c} N_{obs}\\ \vee \\ i=1 \end{array}(\sqrt{{\left(x-x_{obs_{i}}\right)}^{2}+{\left(y-y_{obs_{i}}\right)}^{2}}<Z_{0})$$
  where $N_{obs}$ is the number of obstructions that the MASS needs to avoid in the present state of the ship, ∨ is the symbol “OR”, and $\left(x_{obs},y_{obs}\right)$ is the obstacle position. $Z_{0}$ is the safe encounter distance of the ship.

The safe encounter distance of the ship is related to the size of the ship (length). A large-sized ship will have a long required safety distance.

Both the environmental state set and motion state in the deep Q-learning algorithm are limited, and the actual MASS transportation process is a continuous systematic event. Thus, this study generalizes the activation function as a nonlinear hybrid function:

(5) $$R=\{\begin{array}{cc} 10, & dis_{M-P}(t)=0\\ 2, & s=1\;\mathrm{and}\;(dis_{M-P}(t)-dis_{M-P}(t-1))<0\\ -1, & s=0\\ -1, & s=1\;\mathrm{and}\;(dis_{M-O}(t)-dis_{M-O}(t-1))<0\\ 0, & else \end{array}$$

where s=0 represents the collision between the MASS and obstacle; s=1 represents sailing in a safe area; $dis_{M-P}(t)$ represents the distance between the target point and the MASS at time t; $dis_{M-P}(t-1)$ represents the distance between the target point and the MASS at time t−1; $dis_{M-O}(t)$ represents the distance between the obstacle and the MASS at time t; and $dis_{M-O}(t-1)$ represents the distance between the obstacle and the MASS at time t−1.

### 4.3. Action Selection Strategy

On the one hand, the reinforcement learning system requires online trial and error to obtain the optimal search strategy, namely, exploration; on the other hand, it requires consideration of the entire route planning, so that the expectation of the algorithm to obtain rewards is at a maximum, namely, utilization. It implies that when the search behavior maximizes the action value function, the probability of selecting the action is $1-\varepsilon +\frac{\varepsilon }{\left|A(s)\right|}$ and the probability of selecting other actions is $\frac{\varepsilon }{\left|A(s)\right|}$.

(6) $$\pi (a|s)\leftarrow \{\begin{array}{cc} 1-\varepsilon +\frac{\varepsilon }{\left|A(s)\right|} & \mathrm{if}\;a={\mathrm{argmax}}_{a}Q(s,a)\\ \frac{\varepsilon }{\left|A(s)\right|} & \mathrm{else} \end{array}$$

where π(a|s) represents the navigation strategy in state s by action a. ε∈(0,1] are probabilities of exploration. Q(s,a) is state-action value function of action a after scaling in state s. A(s) is action-value function in state s.

### 4.4. Decision-Making for the Autonomous Navigation of the MASS in an Uncertain Environment

In this section, the abovementioned MASS autonomous navigation decision-making module collects the ship’s own information and environmental status through the sensing layer as input for deep reinforcement learning. Through the system self-learning, the best navigation strategy is finally decided, which makes the cumulative return of MASS in the self-learning process the largest. Once the system is trained, MASS will automatically navigate to avoid obstacles and reach the destination under the command of the autonomous navigation decision-making level.

**Figure 5:**
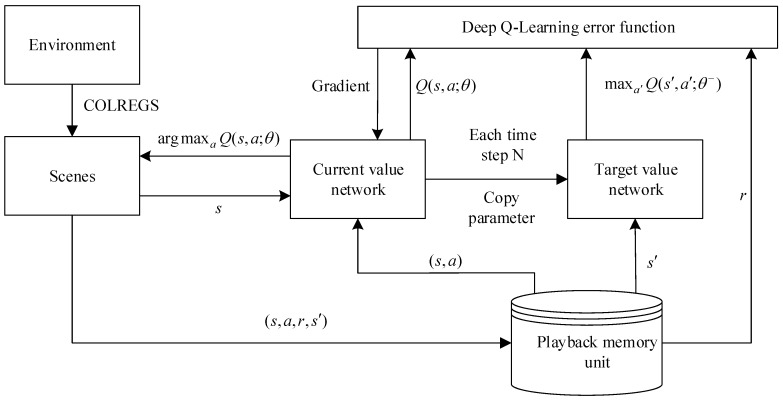
Main flowchart of the autonomous navigation decision-making for MASS.

According to the designed algorithm, first, the MASS is combined with COLREGS to divide the navigation situation into the individual sub-scenarios. Second, the system takes the perceived environmental state information as an input of the current value network and then generates an action based on the current policy through training. It then performs an action to obtain the empirical data and store them in the playback memory unit. Finally, the empirical data are used to update the value function and model parameters until the error is the smallest and the cumulative return value is at a maximum. Figure 5 shows the main flowchart for the high-level driving decisions for MASS.

**Algorithm 1.** DRL for MASS Autonomous Navigation Decision-making
● **Input:**
Start sampling from random state $s_{0}$ and randomly select action. Sampling is terminated at T cycles or the MASS collides. The resulting sample set is S.
Each input in S must be included:
(1) Current states $s_{t}$, (2) action a, (3) return r, (4) the next state after the action $s_{t+1}$, and (5) the termination condition
● **Output:** weights parameter $\omega^{*}$ for DRL
**Require**: ω: a small positive number representing the allowed smallest convergence tolerance; S: the state set; $P(s^{\prime },r|s,a)$: the transition probability from current state and action to next state and reward; γ: the discount factor;
1: Initialize the optimal value function Q(s), ∀s∈S arbitrarily2: **For** episode = 1, *M* **do**3:    **For** t = 1, *T* **do**4:    repeat5:    ω←06:    for s∈S do7:      target q←Q(s)8:      $Q(s)\leftarrow \max_{a}\left[r+\gamma \sum s^{\prime },rP(s^{\prime },r|s,a)Q(s)\right]$9:      ω←max(ω,|q−Q(s)|)10:   until ω<011:   **end for**12: **end for**13: $\pi^{*}(s)\approx argmax_{a}\left[r+\gamma \sum s^{\prime },rP(s^{\prime },r|s,a)Q(s)\right]$


## 5. Simulation and Evaluations

**Figure 6:**
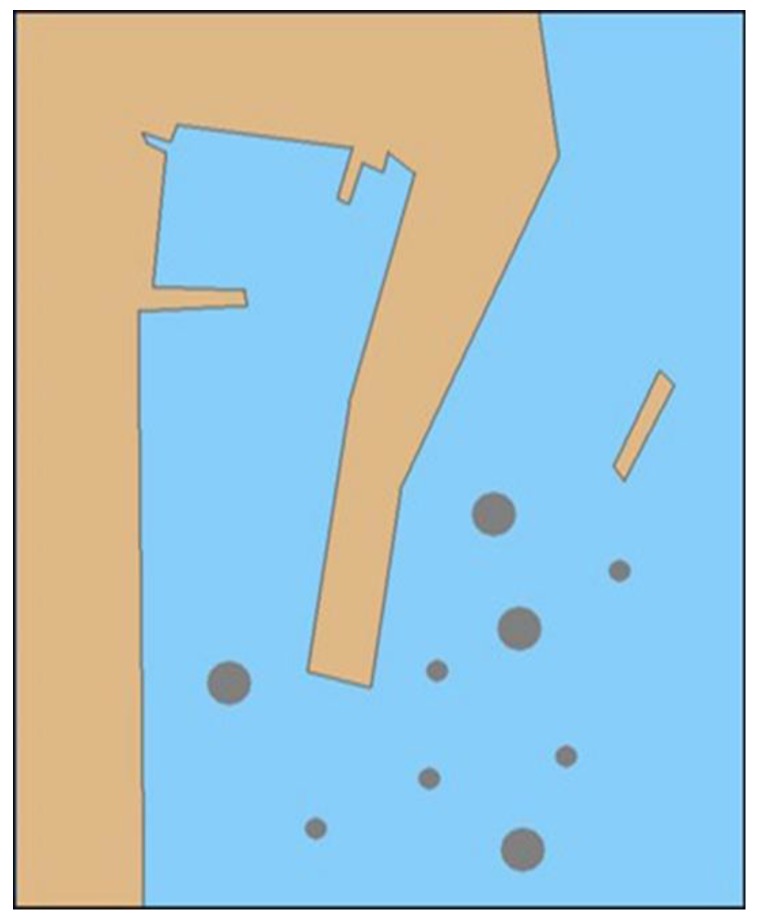
Simulation environment model.

In this section, it is shown that the effectiveness of the autonomous navigation decision-making algorithm for the MASS based on deep reinforcement learning was verified by the case study. This experiment built a two-dimensional (2D) simulation environment based on Python and Pygame. Specifically, the NumPy library and sys, random, and math modules were used for the simulation. In the 2D coordinate system, each coordinate point corresponded to a state of the MASS, and each state could be mapped to each element of the environmental state set. In the simulation environment model, there were two state values for each coordinate point, which were 1 and 0, where 1 represented the navigable area, which is shown as the sea-blue area in the environmental model, and 0 represented the obstacle area, which is shown as a brown and dark gray area in the environmental model. In accordance with the simulation environment model in Figure 6, the 2D map of the state of the simulation environment was simulated, and the obstacles, such as the ship, breakwater, and shore bank were simulated in the environmental model. For the MASS, the location information for these obstacles was uncertain.

### 5.1. Autonomous Navigation Decision-Making Based on DRL

This validation trial section was designed to combine reinforcement learning with deep learning. The goal of the navigation decision-making for the MASS consisted of two parts: the tendency toward the target and obstacle avoidance. If there were no obstacles or few obstacles in the environment, the MASS will randomly select the action to approach the target point with probability $\frac{\varepsilon }{\left|A(s)\right|}$. If the obstacle appeared within the safe encounter distance, the MASS will pass the incentive. The function interacted with the environment to avoid obstacles. Some of the model parameters in the experiment were set as: ω=0.02, γ=0.9, and $v_{0}=8kn$.

**Figure 7:**
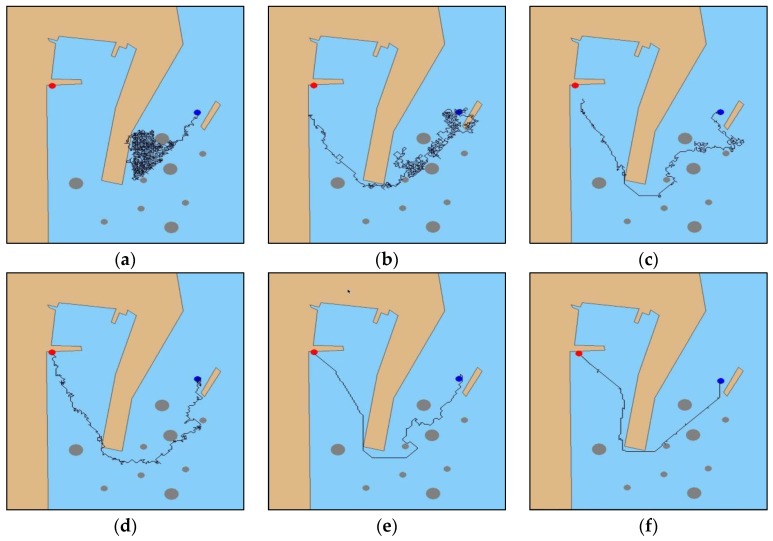
DRL algorithm verification experiment results: (**a**) initial iteration; (**b**) 100th iteration, (**c**) 200th iteration, (**d**) 500th iteration, (**e**) 1000th iteration, and (**f**) 2000th iteration.

The experiment set the initial position (128, 416) and target point (685, 36) of the MASS. As shown in Figure 7a, in the initial iteration, the MASS could not determine the temptation area in the simulation environment and fell into the “trap” sea area in the simulation port pool. As shown in Figure 7b, after 100 iterations, the system gradually planned the effective navigation path, but the collision obstacle phenomenon occurred many times in the process, and the planning navigation path fluctuated significantly. As shown in Figure 7c,d, after 200 to 500 iterations, respectively, the collision phenomenon was gradually reduced, and the planning path fluctuation slowed down. As shown in Figure 7e, all the obstacles were effectively avoided after iterating 1000 times and the planned path fluctuations were weak and gradually stabilized. As shown in Figure 7f, up until the 2000th iteration, the probability of a random search was the smallest, and the system navigated the final fixed path through the decision system to reach the target point.

### 5.2. Improved Deep Reinforcement Learning Autonomous Navigation Decision-making

Although DRL-based MASS navigation behavioral decision-making and path planning in an uncertain environment were realized, the algorithm iteration speed was too slow in the whole experiment, the total iteration time was up to 14 min, and it was trapped in local iterations many times. Affects the applicability and credibility of behavioral decisions. Therefore, there was a need to improve the DRL-based behavioral decision-making algorithm. Therefore, this section describes the addition of an artificial potential field (APF) to improve the DRL and establish an autonomous navigation decision-making-based APF-DRL. To this end, increasing the gravitational potential field was the initial Q value of DRL, avoiding the huge number of calculations in the complex environment, and effectively preventing the MASS from falling into the concave trap in the environment and speeding up the iteration speed of the algorithm.

The DRL algorithm had no prior knowledge of the environment, and all state value functions V(s) in the initial state were equal or completely random. Each step of action a was produced in a random state, i.e., the Markov decision process (MDP) environment state transition probability was equal. For the track decision problem, the return value R was only be changed when the destination was reached or obstacles are encountered. The sparsity of the reward function resulted in an initially low decision efficiency and numerous iterations. Particularly for large-scale unknown environments, there was a large amount of invalid iterative search space.

Combining the APF method to improve the autonomous navigation decision-making based on DRL:

1. Starting point coordinate A and the target point coordinate B were determined. A gravitational potential field with the target point as the potential field center in the initialization state value function was established. The V(s) table was initialized according to the position of the target point as prior environmental information, and the initialized V(s) value was set as larger than or equal to 0.
2. This study conducted a four-layer layer-by-layer search of the environment map. If an obstacle was found, an operation was performed according to v(S(t+1))=−A.
3. The state-action value function table was updated using the environment state value function:
  (7) $$Q(S,A)=r+\gamma V(s^{\prime })$$
4. The MASS explored the environment from the starting point and only considered the state of v(S(t+1))≥0 as an exploitable state. It adopted a variable greedy strategy and updated the state-action value each time it moved. After reaching the target point, this round of iteration ended, and the next round of exploration started from the starting point.

For autonomous navigation decision-making based DRL in complex environments, the action state space is large and iteration speed is slow. When the gravitational field of target point was added as the potential field center to improve the DRL algorithm, the autonomous navigation decision-making of unmanned ships tends to the target point more quickly and iteratively, and the navigation strategies given in each state are directional, whereas a random strategy ensures that it does not fall into a local optimal solution. The dynamic and experimental parameters of the ship were the same as those in Section 5.1.

**Figure 8:**
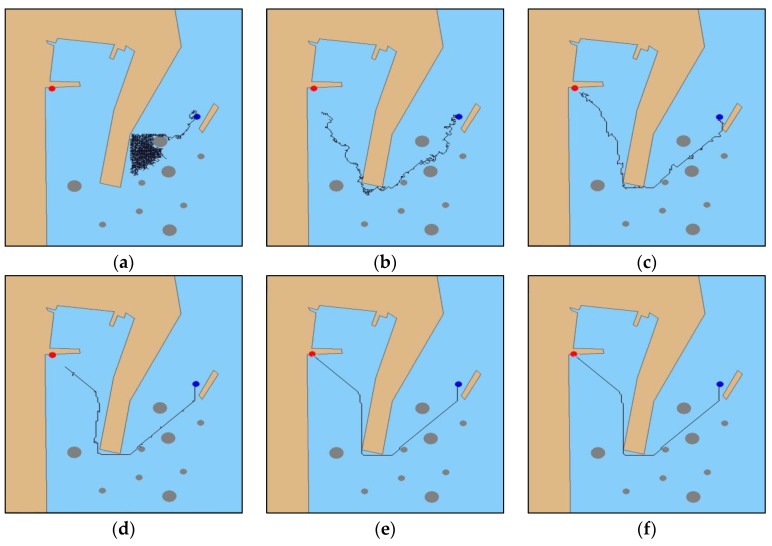
Improved DRL algorithm to verify the experimental results: (**a**) initial iteration; (**b**) 100th iteration, (**c**) 500th iteration, (**d**) 1000th iteration, (**e**) 1500th iteration, and (**f**) 2000th iteration.

The experiment set the initial position (128, 416) and target point (685, 36) of the MASS. In the early stages of the experimental iterations, the MASS collided with the obstacle at different time steps, and there was no collision after the initial iteration in the experiment. The system maneuvered the MASS back to the previous navigation state and re-decided the navigation path planning strategy. Compared with the experiment in Section 5.1, as shown in Figure 8a, in the initial iteration, the MASS fell into a local iteration. As shown in Figure 8b, after 100 iterations, the system first planned an effective navigation path, and the collision obstacle phenomenon occurred multiple times in the process, but the path of the experiment with the same iteration step was shorter than that in Section 5.1. As shown in Figure 8c,d, compared with the Section 5.1 iteration steps, the collision phenomenon was reduced and the path fluctuation was significantly slowed down. As displayed in Figure 8e,f, at the 1500th iteration, the system had completed the high-level navigation decision-making and acquired the optimal navigation strategy until 2000 iterations. The final authenticated publication is available online in reference [24].

### 5.3. Result

**Figure 9:**
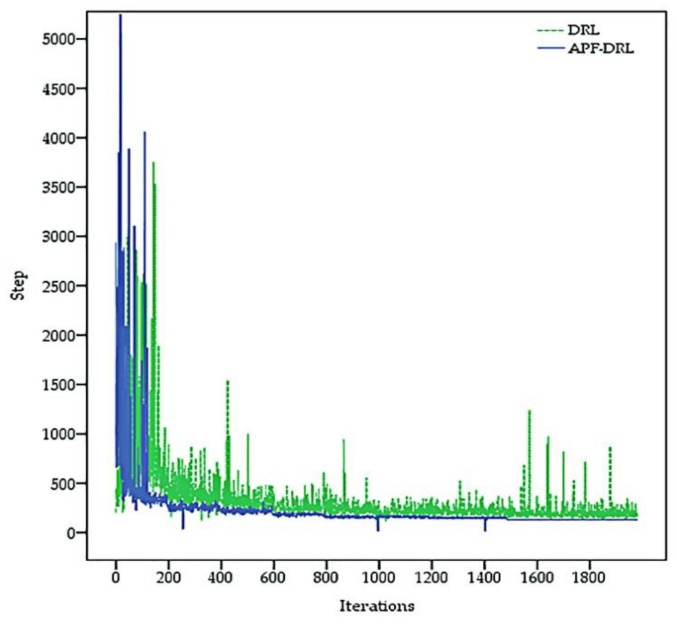
Iterative convergence trend comparison result.

By comparing the verification experiments of the DRL algorithm in Section 5.1 and the APF-DRL algorithm in Section 5.2, the autonomous navigation decision-making-based APF-DRL had a faster iteration speed and a better decision-making ability. Plotting a graph with the training times of the model as the abscissa and the number of steps required to move from the starting point to the end of each iteration as the ordinate allows us to visually demonstrate the training speed and training effect of the two algorithms. The iterative convergence trend comparison presented is shown in Figure 9. The solid blue line represents the iterative trend of the APF-DRL algorithm, and the green dashed line represents the iterative trend of the DRL algorithm. The APF-DRL algorithm did not fall into local iteration after the 500th iteration (step > 500), while the DRL algorithm was iteratively unstable and would still fall into local iteration after the 1500th iteration. The APF-DRL iteration trend had converged by the 1500th iteration, and a fixed path was decided.

Two sets of experimental data were extracted, and the performance of the two navigation decision-making algorithms were compared and analyzed from the aspects of the number of local iterations, large fluctuation iterations (waves more than 300 times), collision rate, optimal decision iteration number, and optimal decision iteration time. As presented in Table 2, compared with the DRL algorithm, the decision algorithm that added the artificial potential field to improve the deep reinforcement learning had fewer local iterations. Moreover, the number of fluctuations and the trial and error rate were reduced. The simulation result shows that the APF-DRL algorithm had a fast convergence rate.

**Figure 10:**
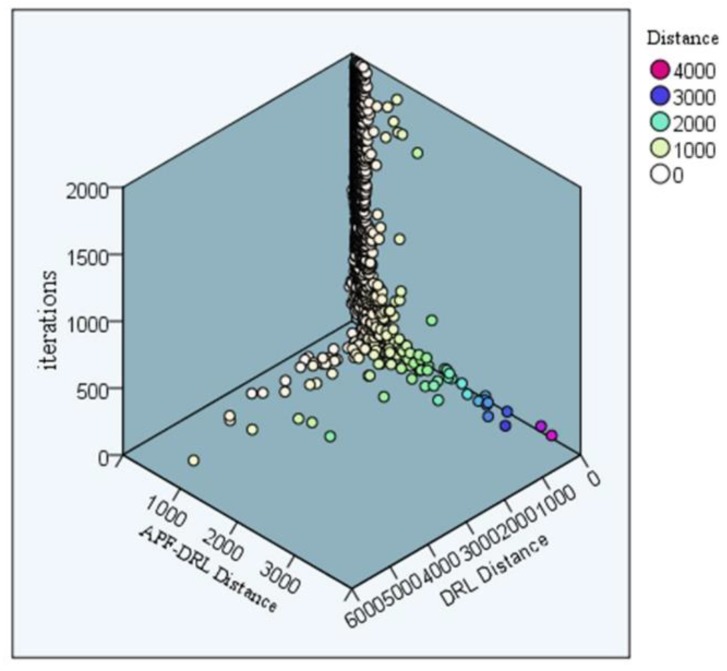
Experimental iteration step scatter plot.

In addition, the step size of each iteration of the two sets of experiments and iterative trends were visually analyzed. Figure 10 displays the comparison of the iterative step size scatter distribution of the two sets of experiments. The color of the scatter in the graph represents the path length of the decision. The DRL-distance was up to 4000, and the APF-DRL algorithm had a maximum iteration step size of 3000, which was much smaller than 4000. Furthermore, the distribution trend of the two sets of scattering points exhibited that the scatter points were almost concentrated on the DRL experimental iteration surface, which indicated that the improved algorithm had a shorter distance for each iteration. Thus, the experimental iterations done by the new procedure were better.

**Figure 11:**
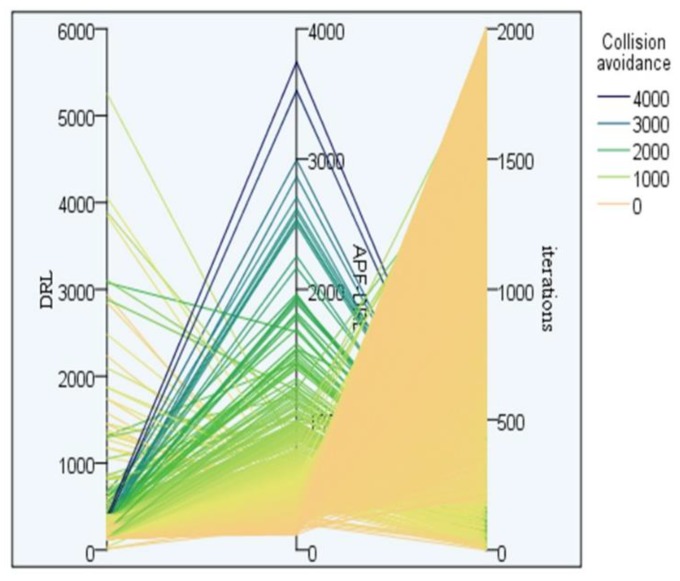
Experimental collision avoidance parallel diagram of the two autonomous navigation decision-making algorithms.

Figure 11 shows the experimental collision avoidance parallel diagram of the two algorithms. The three parallel axes in the figure, from left to right, were DRL avoidances, APF-DRL avoidances, and epochs. The heavier the color, the worse the algorithm learning effect and the slower the convergence. For the APF-DRL algorithm, the avoidance presents a regular distribution, where as the number of iterations increased, the number of collisions decreased, and the self-learning success rate of the algorithm improved. On the contrary, DRL’s collision avoidance iteration diagram is rather messy. It shows that the algorithm’s self-learning ability was unstable.

In summary, by comparing the convergence and decision-making effects of APF-DRL and DRL algorithms from multiple aspects, this study finds that the performance of the models and algorithms based on an artificial potential field to improve deep reinforcement learning was better.

## 6. Conclusions

In this paper, the autonomous navigation decision-making algorithm for a MASS based on the DRL algorithm is proposed. The model was established based on the four basic elements of deep Q-learning. However, the simulation results were not satisfactory, and the easy-to-fall-into local iterations and slow iteration convergence speed problems were apparent. To solve this problem, we added a gravitational potential field centered on the target point, and established an autonomous navigation decision-making algorithm of the MASS based on APF-DRL. In the initial stage of the interaction with the environment, the MASS had little knowledge of the environmental status information, and there were collisions and large fluctuations in the navigation path planning. As the number of iterations increased, the MASS accumulated learning experience, completed the adaptation to the environment, and finally successfully planned the path and reached the target point. In future research, the algorithm still needs significant improvements:

1. The deep Q-learning algorithm based on the Markov decision process could obtain the optimal path through the trial and error algorithm, but its convergence speed was still slower and the number of iterations was large. The first intended improvement involves enhancing the adaptive ability of DRL such that a small number of iterations can be used to learn the correct navigation behavior with only a small number of samples.
2. The strategy functions and value functions in the DRL were represented by deep neural networks, where the networks were poorly interpretable. This unexplained the security problem, which is unacceptable in unmanned cargo ship transportation. The second intended improvement is to improve the interpretability of the model.
3. In the actual voyage, the navigation behavior of the unmanned cargo ship had a complex continuity. In this simulation experiment, only a simple generalization was performed to divide the navigation behavior of the MASS into the eight navigation actions. As such, the third intended improvement direction is to increase the ability of the model to predict and “imagine.”

## Figures and Tables

**Table 1:** MASS ontology model relationship attribute table.

ID	Object Attribute	Domain	Ranges	Comments
1	hasBehindLeftES	Egoship	StaticObstacle	Orientation relationship
2	hasBehindES	Egoship	StaticObstacle	
3	hasBehindRightES	Egoship	StaticObstacle	
4	hasFrontLeftES	Egoship	StaticObstacle	
5	hasFrontES	Egoship	StaticObstacle	
6	hasFrontRightES	Egoship	StaticObstacle	
7	hasLeftES	Egoship	StaticObstacle	
8	hasRightES	Egoship	StaticObstacle	
9	hasFrontED-HO	Egoship	DynamicObstacle	
10	hasFrontED-OT	Egoship	DynamicObstacle	
11	hasFrontED-CR	Egoship	DynamicObstacle	
12	hasBehindED-OT	Egoship	DynamicObstacle	
13	hasFrontLeftED-CR	Egoship	DynamicObstacle	
14	hasFrontLeftED-OT	Egoship	DynamicObstacle	
15	hasFrontRightED-CR	Egoship	DynamicObstacle	
16	hasFrontRightED-OT	Egoship	DynamicObstacle	
17	hasBehindLeftED-CR	Egoship	DynamicObstacle	
18	hasBehindRightED-CR	Egoship	DynamicObstacle	
19	isOnSeapart	Egoship/ObstacleEntity	Seapart	Positional relationship
20	isOnChannel	Egoship/ObstacleEntity	Channel	
21	isOnAnchorage	Egoship/ObstacleEntity	Anchorage	

ES—relationship between the MASS and static obstacles, ED—relationship between the MASS and dynamic obstacles, HO—head-on encounter, OT—overtaking encounter, CR—crossing encounter.

**Table 2:** Experimental data comparison.

Verification Experiment	Trapped into Local Iterations (Times)	Fluctuations > 300 Iterations (Times)	Rate of Collision	Optimal Decision Iterations (Times)	The Iteration Time to Optimal Decision(s)
DRL	56	604	2.24%	2000	859
APF-DRL	29	192	1.16%	1498	442
